# Contact Endoscopic Surface Vascular and Epithelial Morphology in Leukoplakia and Carcinoma of the Vocal Cords: Vascular and morphological changes of vocal folds in leukoplakia and cancer

**DOI:** 10.1007/s12070-023-04183-5

**Published:** 2023-09-20

**Authors:** Pavlos Pavlidis, Vasileios Spyridon Tseriotis, Christopher Matthias, Ioulia Katsikari, Aimilios Chatzinikolaou, Haralampos Gouveris

**Affiliations:** 1 Department of Otorhinolaryngology / Head and Neck Surgery, University Medical Center Mainz, Badralexi 3, Veria, 59132 Mainz, Germany; 2 Laboratory of Clinical Pharmacology, Aristotle University of Thessaloniki, Thessaloniki, Greece; 3 ENT-Clinic, General Hospital Veria, Veria, Greece

**Keywords:** Vocal cords, Capillary, Epithelial, Hyperplasia, Contact endoscopy, Leukoplakia, Squamous cell carcinoma

## Abstract

*Purpose* Leukoplakia is a macroscopic morphological term for thick white or grey mucosal patches that can represent various histologic diagnostic entities ranging from hyperplasia to malignancy. Aim was the study morphology of the superficial mucosa and microvascular network of the vocal cords in patients with suspected glottic squamous cell carcinoma (SCC) using contact endoscopy (CE). *Material and Methods* Seventy-nine patients (21 female, 58 male), with a mean age of 57.5 years ± 7.12 (range, 32–73 years), were prospectively enrolled and evaluated. Of these patients, 58 had leukoplakia (Group A/41 males and 17 females, with a mean age of 53.7 years ± 6.65), and 21 (Group B/ 17males and 4 females/ with a mean age of 60.5 years ± 6.04) had malignant lesions (pT1, *n* = 6; p T2, *n* = 8; pT3, *n* = 8; Group B), as proven by the results of the histological examination. Further, 79 non-smokers (control group—group C) were studied. CE imaging findings were classified into five types (I to V) based on the features of the mucosal intra-epithelial capillary loops. CE findings were correlated to the histologic findings. A separate analysis involving smoking status was done. *Results* The CE-based intraepithelial papillary capillary loop classification score was strongly correlated with the histological findings. Age was strongly associated with both malignancy and bilateral involvement. Smoking habits didn’t significantly differ between patients with unilateral and bilateral SCC. *Conclusions* CE imaging of the vocal cord mucosal capillaries may be useful for the early detection of glottic SCC and pre-cancerous lesions.

## Introduction

Leukoplakia is a macroscopic morphological term for thick white or grey mucosal patches that can represent various histologic diagnostic entities ranging from hyperplasia to malignancy [1–4]. Endoscopic (white light, WL) examination combined with stroboscopy is currently the standard core approach for detecting and assessing vocal cord leukoplakia (VFL) or other vocal cord lesions [5–7]. Contact endoscopy (CE) was originally described by Hamou in 1979 as a technique for the visualization of cervical and uterine epithelial cells for screening and diagnosis of cervical and uterine pathology [3]. Andrea et al. used CE to examine the vocal cords and nasal mucosa in the 1990s [8, 9]. Thereafter CE use has been reported in the diagnosis of mucosal lesions of the oral cavity [6], oropharynx, hypopharynx [7], nose [8], nasopharynx [9], recurrence of ear cholesteatoma [10], per operative identification of parathyroid glands [11], comparison of fungiform papillae during aging [11], in diabetes mellitus [12], in Bell’s palsy & herpes zoster [13], after the transaction of the chorda tympani facial nerve branch [12], in smokers and after smoking cessation [14].

The goal of this study was to evaluate distinct vascular patterns in patients with VFL and glottic squamous cell carcinoma (SCC) using contact endoscopy and investigate whether there are any correlations between the CE findings and the standard histologic findings in the mucosa of the vocal cord in these patients.

## Materials and Methods

### Clinical Epidemiologic Data

Seventy-nine patients (21 female, 58 male), with a mean age of 57.5 years ± 7.12 (range, 32–73 years), were prospectively enrolled and evaluated.

Of these patients, 58 had leukoplakia (Group A), and 21 had malignant lesions (pT1, n = 6; p T2, *n* = 8; pT3, *n* = 8; Group B), as proven by the results of the histological examination. Regarding the patients with malignancies, 8 had bilateral lesions, and thus 29 SCC lesions were studied and detected with CE. Between patients with leukoplakia and no malignancy, there was a total of 77 lesions. Therefore, a total of 106 lesions (29 malignancies/squamous cell carcinoma and 77 non-SCC) were found. Concerning the leukoplakia-patients, by 29 of them a low-grade dysplacia has been observed, and the rest of them suffered from a high-grade one. By the tumor-suffering patients, all of them had a high grade SCC.

In Table 2, demographic features, such as smoking status and age, of patients with and without SCC are depicted and compared.

For this study, we have additionally examined 79 non-smokers (control group–group C), who received total anesthesia for surgeries for non-otolaryngological diseases, such as hernias or other abdominal surgeries.

The study protocol was reviewed and approved by the Institutional Review Board ( Papanikolaou General Hospital, Thessaloniki, Nr. 72–2,204,202). All participants provided informed consent for participation in the study after being instructed about the aim and the procedures of the study. The procedures were in accordance with local data protection guidelines and legislation and according to the principles of the Helsinki Declaration for studies in humans.

### Contact Endoscopy

For contact endoscopy the Andrea-Dias Contact Micro Laryngoscope (with HOPKINS Straight Forward Telescope 0° and 30°, with diameter 5.5 mm, length 23 cm, magnification 60 × and 150 ×); a 3-chip camera (Tricam SL II); a Xenon 175 Watt light source and a video recording system have been used, all manufactured by Karl Storz (Tuttlingen, Germany).

Vascular patterns were studied before staining because the staining dye causes a loss of transparency of the vocal cord mucosa making the blood vessels invisible. Epithelial cellular architecture was studied after staining the mucosal surface with 1% methylene blue which imparts a dark blue color to the nucleus and a light blue color to the cytoplasm. The excess stain has been removed by washing the area with a copious amount of saline using suction and irrigation. Methylene blue is non-toxic, and the staining is reversible [8, 9, 11

Endoscopically guided biopsy of laryngeal lesions was also performed under general anesthesia; tissue specimens were fixed in 10% formalin for histological analysis [10, 11]. The recorded CE findings were examined by two experienced professionals (PP and VST), who evaluated the pictures separately before discussing the results together. Both of them were blinded to the histological results at the time of assessment of the CE findings. The interrater reliability was calculated with the use of the Kappa test and was equal to 0.89 (Cohen’s kappa statistic).

### Morphological Types of the Surface of the Vocal Cords

The morphological types of vocal cord leukoplakia assessed by preoperative rigid laryngoscopy were categorized as flat and smooth, elevated and smooth, and rough type [11, 12].

These definitions were based on the following morphological pattern categories, as depicted in Table 1.

**Table 1:** The morphological pattern categories, used for the classification of our findings

Morphologic pattern Categories	
Flat and smooth type: Surface: smooth; margin: lesion without raised margins, being continuous with the surrounding mucosa; Texture: homogeneous, regular, the lesion with even coloration	
Elevated and smooth type: Surface: smooth; Margin: lesion with raised margins, sharply demarcated from the surrounding mucosa; Texture: homogeneous, regular, the lesion with even coloration	
Rough type: Surface: wrinkled, corrugated; Margin: lesion with raised margins, sharply demarcated from the surrounding mucosa; Texture: non-homogeneous, irregular, the lesion with uneven coloration and is usually accompanied with erosion or ulceration	

### Patterns and Changes

The Ni categorization was used for our research.^16^ Intraepithelial capillary loop alterations seen on CE can be categorized into five categories (I to V) according to this classification. Intraepithelial papillary capillary loops are nearly inconspicuous in type I, while oblique and arborescent capillaries of small diameter are discernible. The intraepithelial papillary capillary loops are nearly invisible in type II, while the diameter of the apparent oblique and arborescent capillaries is increased. The mucosa is white in type III, and the intraepithelial papillary capillary loops are invisible; if the white patch is thin, the oblique and arborescent vessels can be seen indistinctly, but if the white patch is thick, the vessels are obscured. The mucosal intraepithelial papillary capillary loops appear as scattered, small, dark brown spots in type IV, with a relatively regular arrangement and low density; the capillary terminals are bifurcated or slightly dilated, and the intraepithelial papillary capillary loops appear as scattered, small, dark brown spots; the oblique and arborescent vessels are usually not visible [13].

**Type V** changes are subdivided into **types Va, Vb,** and **Vc** according to the shape, regularity, and distribution of vessels. In type Va, intraepithelial papillary capillary loops are significantly dilated and of relatively high density, and appear to be solid or to have hollow, brownish, speckled features and various shapes [13]. In type Vb, the intraepithelial papillary capillary loop itself is destroyed, with its remnants presenting in a snake-, earthworm-, tadpole- or branch-like shape, and the microvessels are dilated, elongated, and ‘woven’ in appearance. In type Vc, the lesion surface is covered with necrotic tissue, and the intraepithelial papillary capillary loops present as brownish speckles or tortuous shapes of uneven density which are irregularly scattered on the tumor surface [13].

**Fig. 1:**
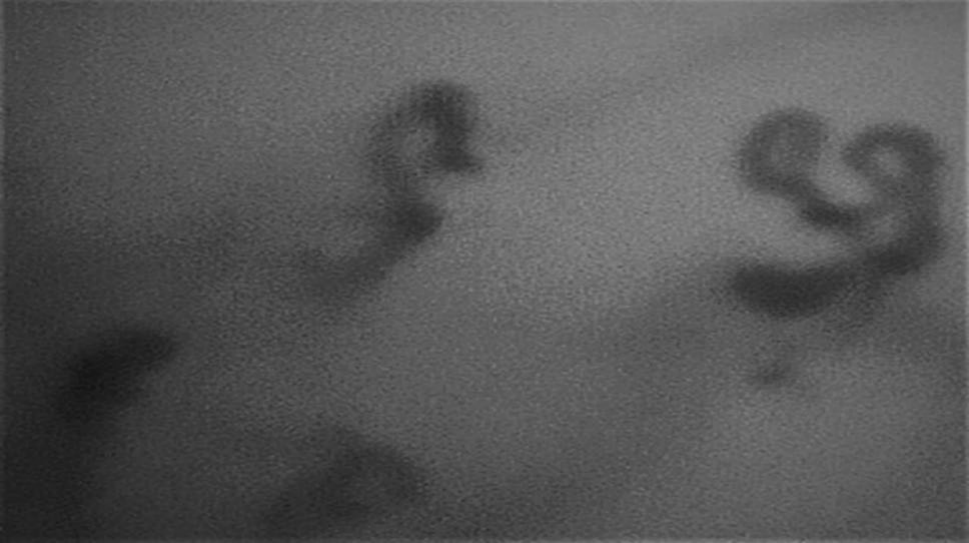
The images depict type I, II, and III patterns of vascularization respectively. In type I, the intraepithelial papillary capillary loops are almost invisible; oblique and arborescent small-diameter vessels can be seen. In type II, the intraepithelial papillary capillary loops are also almost invisible, but the diameter of the observed oblique and arborescent vessels is enlarged. In type III, the mucosa is whitish and the intraepithelial papillary capillary loops cannot be seen; if the whitish patch is thin, the oblique and arborescent vessels may be seen indistinctly, but if the whitish patch is thick the vessels will be obscured.

**Fig. 2:**
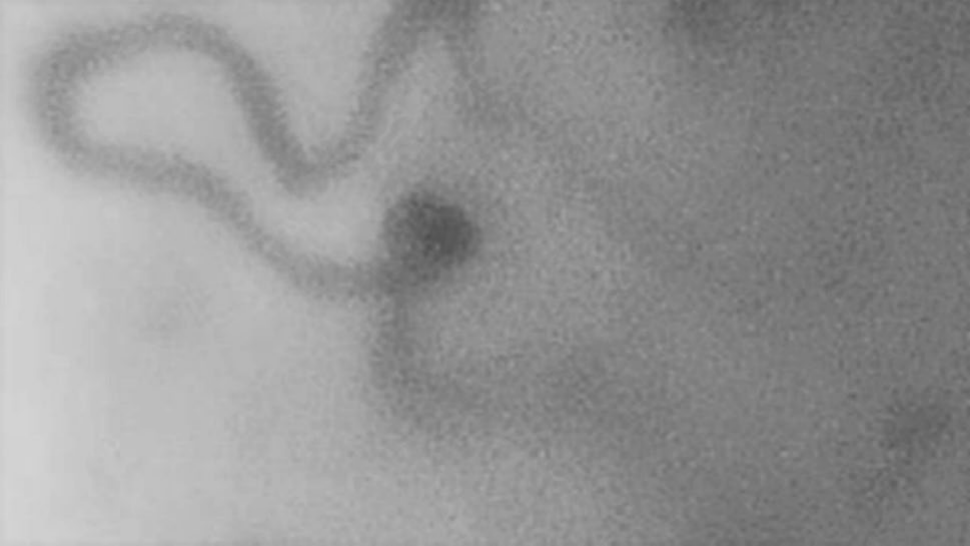
The images depict type I, II, and III patterns of vascularization respectively. In type I, the intraepithelial papillary capillary loops are almost invisible; oblique and arborescent small-diameter vessels can be seen. In type II, the intraepithelial papillary capillary loops are also almost invisible, but the diameter of the observed oblique and arborescent vessels is enlarged. In type III, the mucosa is whitish and the intraepithelial papillary capillary loops cannot be seen; if the whitish patch is thin, the oblique and arborescent vessels may be seen indistinctly, but if the whitish patch is thick the vessels will be obscured.

**Fig. 3:**
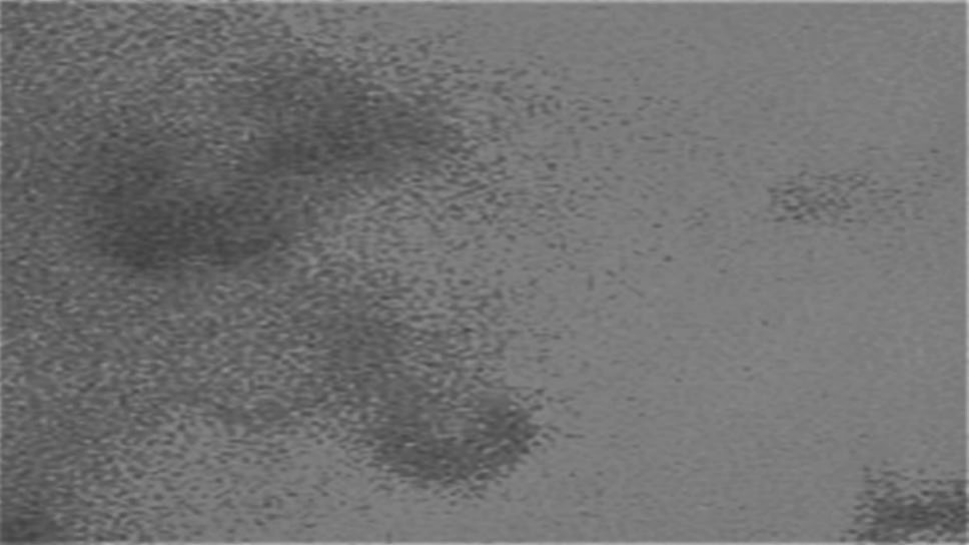
The images depict type I, II, and III patterns of vascularization respectively. In type I, the intraepithelial papillary capillary loops are almost invisible; oblique and arborescent small-diameter vessels can be seen. In type II, the intraepithelial papillary capillary loops are also almost invisible, but the diameter of the observed oblique and arborescent vessels is enlarged. In type III, the mucosa is whitish and the intraepithelial papillary capillary loops cannot be seen; if the whitish patch is thin, the oblique and arborescent vessels may be seen indistinctly, but if the whitish patch is thick the vessels will be obscured.

According to the shape, regularity, and distribution of vessels, type V changes are split into types Va, Vb, and Vc. Intraepithelial papillary capillary loops in type Va are highly dilated and of relatively high density, appearing solid or hollow, brownish, speckled, and of varied shapes.^16^ The intraepithelial papillary capillary loop is disrupted in type Vb, with remains resembling a snake, earthworm, tadpole, or branch, and microvessels that are dilated, elongated, and 'woven' in appearance. The lesion surface is coated with necrotic tissue in type Vc, and the intraepithelial papillary capillary loops appear as brownish speckles or sinuous shapes of uneven density spread irregularly on the tumor surface [12–14]. The various type categories are depicted in Figs. 1, 2, and 3.

### Histologic Examination

All the tissues were processed for pathological testing on a standard basis. The same pathologist, who was blinded to the CE findings, evaluated and graded histologically graded formalin-fixed and paraffin-embedded slides independently. Squamous cell hyperplasia with non-dysplasia, mild dysplasia, moderate dysplasia, severe dysplasia, carcinoma in situ, and squamous cell carcinoma were all assessed histologically according to the World Health Organization's (WHO) 2017 guidelines [15]. The WHO 2017 classification is a two-tier system. Laryngeal precursor lesions are classified as low-grade dysplasia (previous categories squamous hyperplasia, mild dysplasia), and high-grade dysplasia (previous categories of moderate and severe dysplasia, carcinoma in situ). Carcinoma in situ is distinguished from high-grade dysplasia, showing features of conventional carcinoma [15].

### Statistical Analysis

Parameters were evaluated using jamovi software (Version 1.6, Sydney, Australia from www.jamovi.org). A p-value less than 0.05 was considered statistically significant for all analyses. Independent samples t-test, Mann–Whitney U test, and Chi-square test were used for basic characteristics’ comparisons between male and female patients’ features (age, years of smoking, number of cigarettes/day) as well as for comparisons between patients with unilateral or bilateral lesions and patients with or without histologically confirmed malignancies. The association between the appearance of the surface of the vocal cords and the vascular patterns was examined in both groups of leukoplakia and glottic cancer, as well as in healthy vocal cords using the Chi-square test or Fisher’s exact test (if expected counts were less than 5). The association between the healthy cord’s vascularization pattern and the presence or absence of malignancy in the contralateral cord was investigated using the Chi-square test or Fisher’s exact test.

## Results

Age was strongly associated with both malignancy and bilateral involvement, since in our studied cohort SCC lesions were much more prevalent in older rather than in younger participants (*t* = −4.11, *p* < 0.001). Participants with bilateral lesions were significantly older than those with unilateral disease (*t* = −2.06, *p* = 0.043). The grade of dysplasia by the patients (low or high) did not have any effect to the results.

The number of cigarettes smoked daily was significantly higher in the malignancies group compared to patients with no malignancy (U = 262, *p* < 0.001).

Regarding basic characteristics, there was no difference in age, number of cords affected, cigarettes smoked per year, and years smoking between male and female patients.

A Fisher exact test was performed to examine whether the results of intraepithelial papillary capillary loop classification using CE imaging and the morphological types of the lesions could be associated. There appears to be statistical significance, *p* < 0.001, suggesting that in general, there is an association between the macroscopic changes observed on the surface of the vocal folds and the method of CE (Table 2). Thus, “flat and smooth” or “elevated and smooth” vocal cords are more likely to be classified as type I, II, or III as regards vascular categorization, whereas the “rough type” correspond to vascular patterns IV and V. Furthermore, as it can be seen in Fig. 1, both methods were able to detect histologically confirmed malignancies.

**Table 2:** Comparison of demographic features between patients with malignant and non-malignant lesions. Information on smoking status (cigarettes per day and years smoking) and age in different types of vascular classification is also provided

Basic Characteristics				
	Total	Patients with malignant lesions	Patients with non-malignant lesions	
Total number of subjects	79	21	58	
Bilateral lesions	27	8	19	
Sex	58 males, 21 females	17 males, 4 females	41 males, 17 females	*P* = *0.362*
Age (years), mean ± SD	57.5 ± 7.12	60.5 ± 6.04	53.7 ± 6.65	*P* = *0.001*
Cigarette/day, mdn (IQR)	17 (23–12)	24(28–18)	13 (18–12)	*P* = *0.001*
Years smoking, mdn (IQR)	30.1 (37–25)	32 (35–28)	34 (37–25)	*P* = *0.472*

A Fisher’s exact test was used indicating an agreement between the two methods regarding the categorization of vocal cord lesions (*p* < 0.001). fs = flat and smooth type; es = elevated and smooth type; r = rough type.

Lastly, we also wanted to test if there was a specific pattern between the vascularization type of the healthy vocal cord and the presence of malignancy in the contralateral cord of these patients. We used a Fisher’s exact test, which showed statistical significance (*p* < 0.001), suggesting that, as seen in Table 3, healthy cords with early stages of vascular classification (mostly type I) were more likely to belong to patients who have leukoplakia or non-malignant contralateral vocal cords. On the other hand, healthy cords of type III or IV were more likely to be associated with contralateral malignancies (Table 4).

**Table 3:** Association of vascular classification in the healthy vocal cord with the possibility of malignancy in the contralateral vocal cord

Vascular Classification								
Morphological Classification	I	II	III	IV	Va	Vb	Vc	Total
es	0	7	25	5	0	0	0	37
	0%	6.7%	23.8%	4.8%	0%	0%	0%	35.2%
fs	4	11	6	4	0	0	0	25
	3.8%	10.5%	5.7%	3.8%	0%	0%	0%	23.8%
r	0	3	4	8	11	16	1	43
	0%	2.9%	3.8%	7.6%	10.5%	15.2%	1%	41%
Total	4	21	35	17	11	16	1	105
	3.8%	20%	33.3%	16.2%	10.5%	15.2%	1%	100%

**Table 4:** The vascular classification of the patients, combined with that of the morphological one

Vascular Classification							
Morphological Classification	I	II	III	IV	Va	Vb	Total
fs	29	8	0	0	0	0	37
	58%	16%	0%	0%	0%	0%	74%
es	0	0	9	2	1	1	13
	0%	0%	18%	4%	2%	2%	26%
Total	29	8	9	2	1	1	50
	58%	16%	18%	4%	2%	2%	100%

## Discussion

We provide evidence that vocal cord squamous cell carcinoma is associated with a single abnormal vascular pattern on the epithelial surface of the vocal cords, whereas leukoplakia may be associated with either normal or various abnormal vascular patterns. In addition, in patients with leukoplakia, the cumulative time spent smoking (in years) had a detrimental impact on the surface and vascularization of the vocal cords.

Capillaries in the superficial lamina propria, smaller arteries, and veins, as well as arterioles and venules in the deeper layers, characterize the vascular microanatomy of human vocal cords. Arterioles and venules have direct vascular anastomoses [16].

Under rigid laryngeal endoscopy, vocal cord leukoplakia presents as a white or grayish confined patch, distributed granule, or verrucous structure. It may have one or more localizations [10]. Leukoplakia is a chameleon-like epithelial transformation that can range from benign thickening to malignant tumors. As a result, the name "leukoplakia" is insufficient to characterize the lesion's histological identity [15–18].

There are previous reports on a tissue-specific classification of vascular changes associated with laryngeal leukoplakia. According to former reports, age, non-homogenous lesion texture, and the existence of hyperemia are independent predictors of malignancy [18–20]. These reports support the findings of the present study to a certain extent because they also provide evidence that age and lesion texture may predict prognosis. However, the impact of age on leukoplakia lesions has not been extensively explored. Our study provides preliminary evidence that age may be related to the development of this disease. Moreover, a further novelty of the present study is that a very detailed study on the lesion texture has been conducted. Leukoplakia lesions have traditionally been divided into two categories from their appearances which were individually homogenous and heterogeneous in many reports [21].

Although new endoscopic tools, such as narrow-band imaging, optical coherence tomography, and contact endoscopy have been developed to improve the diagnosis of vocal cord leukoplakia, WL laryngoscopy is the usual standard of care in clinical practice [22–25]. The ability of rigid or flexible laryngoscopy to visualize and characterize lesions of vocal cords continues to improve.

Many researchers have reported high efficacy of CE inthe diagnosis of mucosal lesions not only of the larynx but in other sites of head and neck mucosal surfaces as well [10–13, 26]. These results have been obtained by taking the histopathological examination as the gold standard. The technique of CE has definite advantages and limitations. Contact endoscopy enables visualization of tumor margins, dysplasia, and normal epithelium, thus offering the possibility of more precise complete removal of laryngeal lesions in a single sitting. Along with in vivo studies, contact endoscopy can also be used to analyze the excised segment of the lesion and hence ensure whether the lesion has been completely resected. The grade of dysplasia is indicated by the impaired nucleus/cytoplasm ratio, nuclear hyperchromasia, and variation in the number and appearance of the nucleoli [25, 27].

Of course, there are limitations in the use of CE, which should be also considered in the interpretation and validation of the results of the present study. Two inherent limitations are the inability to detect very early dysplasia and the inability to differentiate carcinoma in situ from invasive carcinoma.

## Conclusion

Vascular changes may play an important role as one of the most prominent features in the endoscopic work-up of vocal cord lesions. Further validation of our preliminary findings, especially in combination with further standardized morphologic endoscopy findings (together with macroscopic appearance, mucosal vibration, vocal cord stiffness, and others) should be undertaken to increase the reliability of pre- and intraoperative diagnosis of leukoplakia and malignant glottic lesions.

## Data Availability

The data presented in this study are available at reasonable request from the corresponding author. The data are not publicly available due to ethical/privacy restrictions.
